# Fermentation of *Rubus dolichocarpus* juice using *Lactobacillus gasseri* and *Lacticaseibacillus casei* and protecting phenolic compounds by *Stevia* extract during cold storage

**DOI:** 10.1038/s41598-024-56235-7

**Published:** 2024-03-08

**Authors:** Mohammad Mahdi Karimkhani, Abdollah Jamshidi, Mahmoud Nasrollahzadeh, Mohammad Armin, Seid Mahdi Jafari, Tayebeh Zeinali

**Affiliations:** 1 Department of Food Hygiene and Aquaculture, Faculty of Veterinary Medicine, Ferdowsi University of Mashhad, Mashhad, Iran; 2 Department of Chemistry, Faculty of Science, University of Qom, 37185-359 Qom, Iran; 3 Department of Agronomy, Sabzevar Branch, Islamic Azad University, Sabzevar, Iran; 4 Department of Food Materials and Process Design Engineering, Gorgan University of Agricultural Sciences and Natural Resources, Gorgan, Iran; 5 Halal Research Center of IRI, Iran Food and Drug Administration, Ministry of Health and Medical Education, Tehran, Iran; 6 Department of Nutrition and Food Hygiene, School of Health, Social Determinants of Health Research Center, Birjand University of Medical Sciences, Birjand, Iran

**Keywords:** *Black raspberry juice*, *Lactobacillus gasseri*, *Lacticaseibacillus casei*, *Stevia* extract, High hydrostatic pressure, Biochemistry, Chemical biology, Microbiology

## Abstract

This study aimed to investigate the biological activities of *Lactobacillus gasseri* SM 05 (*L. gasseri*) and *Lacticaseibacillus casei* subsp. *casei* PTCC 1608 (*L. casei*) in the black raspberry (*Rubus dolichocarpus*) juice (BRJ) environment, and also the anti-adhesion activity against *Salmonella typhimurium* (*S*. *typhimurium*) in fermented black raspberry juice (FBRJ). Results showed significant anti-adhesion activity in Caco-2 epithelial cells. In the anti-adhesion process, lactic acid bacteria (LAB) improve intestinal health by preventing the adhesion of pathogens. Adding LAB to BRJ produces metabolites with bacteriocin properties. Major findings of this research include improved intestinal health, improved antidiabetic properties, inhibition of degradation of amino acids, and increase in the nutritional value of foods that have been subjected to heat processing by preventing Maillard inhibition, and inhibition of oxidation of foodstuff by increased antioxidant activity of BRJ. Both species of *Lactobacillus* effectively controlled the growth of *S*. *typhimurium* during BRJ fermentation. Moreover, in all tests, as well as Maillard's and α-amylase inhibition, *L. gasseri* was more effective than *L. casei*. The phenolic and flavonoid compounds increased significantly after fermentation by both LAB (*p* < 0.05). Adding *Stevia* extract to FBRJ and performing the HHP process showed convenient protection of phenolic compounds compared to heat processing.

## Introduction

Berries are edible and juicy fruits that have significant biological properties such as antioxidant and anti-inflammatory characteristics. The berries’ compounds are also very useful for the skin. It is a remarkable feature of these fruits that contain vitamin C, and this vitamin greatly prevents cataracts, arthritis, and macular degeneration^1^. Among the fruits of berry, Raspberries, in terms of vitamin C, are very rich and prevent colds and boost the immune system^2,3^. In some previous studies due to the presence of carotenoids^4^ and α-linolenic acid, tocopherols, linoleic acid and finally oleic acid in raspberry, this fruit is known as having health promotion properties^5,6^. The compounds that protect cells against oxidation are phenolic and flavonoid compounds (resveratrol and apijanine and routine, etc.) as well as anthocyanins, ellagitannins, lignans and tannins in this fruit^7^. Phenols in raspberry also prevent disorders such as Alzheimer, obesity, Parkinson and diabetes^8,9^. Dark or black berries have more antioxidants than red berries and these high antioxidants have a direct relationship with the phenolic and flavonoid compounds of these fruits^10^.

LAB were seen and described in milk for the first time in 1780. They utilize fermentative carbohydrates to form lactic acid^11,12^. The benefits of lactic fermentation include controlling some cancers, preventing the development of intestinal infections, improving the nutritional value of some foods, and preventing the increase in cholesterol levels in the body. Furthermore, as fermentation in dairy products leads to the reduction of lactose sugar, and it could also be useful for some people who have an intolerance to lactose^13^.

Among the LABs, *Lactobacillus gasseri* and *Lacticaseibacillus casei* have therapeutic indications; for example, a study reported *L. gasseri* improved stress and anxiety in healthy people as well as sick people with intestinal problems^14^. Moreover, *L. gasseri* BNR17 was effective in weight loss^15^ and *L. gasseri* G098 reduced colitis caused by dextran sodium sulfate^16^ and prevented the growth of bacteria and viruses in the vaginal environment^17^. *L. casei* variety *rhamnosus* has been used to treat or prevent diarrhea^18^. It is also reported that if foods containing *L. casei*-01 are consumed continuously, it prevents increasing blood pressure and blood sugar^19^.

Pathogens are invaders that attack the cells of the host body. Pathogens have a wide range of characteristics, some of them attack a single host and others can attack a wide range of hosts^20^. *Salmonella enterica* serovar *typhimurium* is a primary intestinal pathogen that infects animals as well as humans. The infection begins by eating contaminated food or water so that salmonellae colonize the intestinal epithelium and cause gastrointestinal diseases. *Salmonella* has especial clinical importance in both developed and developing countries and is the cause of most common diarrheal diseases transmitted through food. Several outbreaks of *Salmonella* serovars are reported annually as *S. enterica* serovar *typhimurium* and *S. enterica* serovar enteritidis as the most common etiological agents^21^.

There are important reasons that the combination of these two bacteria with BRJ has been used:

1. According to the nutritional grade of the two bacteria used, *L. gasseri* has been isolated from the fermentation of camel milk. *L. casei* has also been isolated from Kraft Swiss cheese. Also, by improving the antioxidant properties of BRJ, the harmful effects of oxidation in the body and food can be prevented.
2. High medicinal properties of these two bacteria: these two important LABs potentially have functional and valuable properties that have high therapeutic and medicinal potential and so far, it has not been investigated in BRJ, and its combination with BRJ can improve health by producing useful metabolites at the end of fermentation and produce an important functional drink in terms of health promotion because it can be useful and effective against harmful bacteria, such as salmonella, which are involved in intestinal diseases.
3. Due to the compatibility of these bacteria with the food environment and good growth in BRJ and the high fermentation power of simple sugars and nutrients in fruit juice, combining each of the LABs with BRJ produces a new product and this inoculation by increasing phenolic and flavonoid content in BRJ improves the functional and nutritional properties of BRJ. Maintaining these properties over time using *Stevia* extract is another goal of this research, and the *Stevia* plant has the following characteristics.

*Stevia* rebaudiana Bertoni is one of the two species that produces glycosides in addition to its sweetening properties^22^. This plant has also many biological and therapeutic properties, including anti-oxidant, anti-diabetic, anti-obesity, anti-tumor, etc.^23^. Due to the reduction of sugar in the fermentation process by two LABs and also, considering the high sweetness properties of the *Stevia* plant and its therapeutic properties, the extract of this plant was used along with different HHP and VAT pasteurization methods.

Therefore, this study was performed to investigate the antagonistic and anti-adhesion effects of the mentioned *Lactobacillus* strains on *S. typhimurium* during the fermentation of BRJ. The biological activities of FBRJ, including inhibition of the Maillard reaction, and antioxidant and antibacterial properties were also investigated. The stability of the phenolic and flavonoid compounds and the antioxidant strength of the samples were examined during 28 days of storage in the presence of *Stevia* extract.

## Results and discussion

### Viable cells and pH

**Figure 1:**
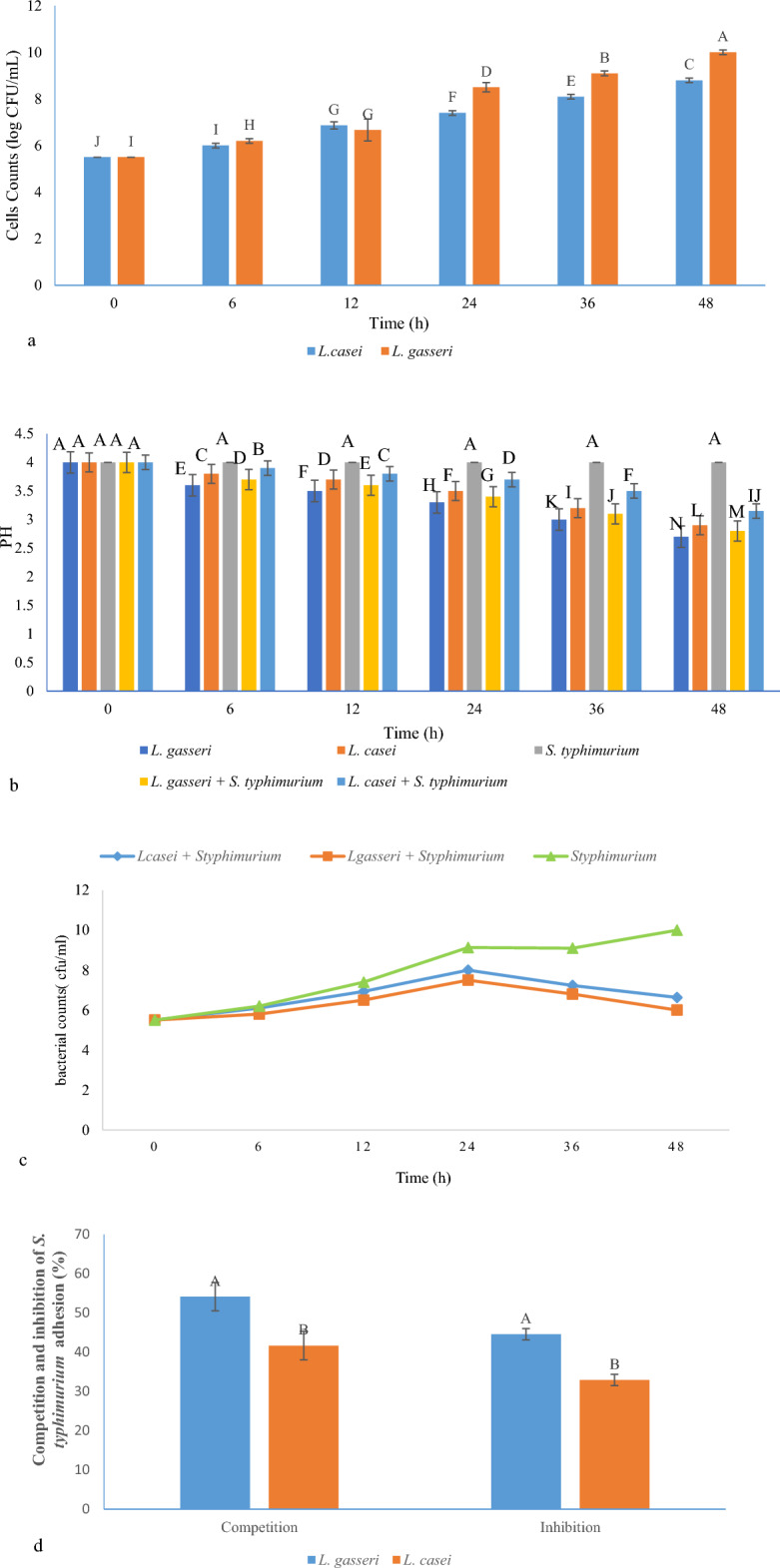
(**a**) Changes in cell counts of *Lactobacillus* strains in FBRJ; (**b**) changes in pH of BRJ during fermentation for 48 h; (**c**) changes in cell counts of *S. typhimurium* in various BRJ samples; (**d**) anti-adhesion assay (inhibition and competition of *S. typhimurium* in the presence of *L. gasseri* and *L. casei* strains) (different capital letters show the significant difference (p ≤ 0.05).

Figure 1a,b show the growth trend of bacteria and pH changes in FBRJ. Initially, the bacteria were inoculated with 5.5 log CFU/mL in BRJ. On the second day of fermentation, the fermentation process was performed slowly and steadily. But, on the first day of fermentation, bacterial production was also much higher than the second day of fermentation. For *L. gasseri* its value reached 10 log CFU/mL and for *L. casei* it was 8.8 log CFU/mL at the end of 48 h of fermentation. The pH was also four at the beginning of fermentation and during the fermentation of BRJ containing both LABs, pH decreased to 2.7 for *L. gasseri* and 2.9 for *L. casei*. In 2021, Li et al. reported that different strains of *L. fermentum* were inoculated with 7.5 log CFU/mL in blueberry juice^24^. By the end of 48 h of fermentation, the number of LAB in all subspecies had increased by more than 35% and some bacteria had reached more than 10 log CFU/mL^24^. In this study, a more than 81% increase in bacterial population was observed for *L. gasseri* and a 60% increase was observed for *L. casei*.

Two strains of *Lactobacillus* were used in BRJ against *S. typhimurium*. As shown in Fig. 1b*, L. gasseri* was effective in reducing pH compared to other treatments that pH reached 3.3 after 24 h and at the end of the fermentation process was 2.7, while pH reduction in fruit juice containing *S. typhimurium* did not occur during the experiment. It is noteworthy that due to the activity of each lactic bacterium alone in BRJ, the pH rate was lower than when each lactic bacterium was used in combination with *S. typhimurium*. Of course, this indicates acid production by LAB.

As shown in Fig. 1c, the changes in *S. typhimurium* count along with any of the tested *Lactobacillus* are different compared to BRJ and *S. typhimurium* without LAB. During the fermentation period where the LAB and *S. typhimurium* are present, the number of pathogenic bacteria increases in the first 24 h of fermentation. But after 24 h, the number of *S. typhimurium* decreases until the end of the fermentation process. When only pathogenic bacteria without LAB were present in BRJ, until the end of 48 h of fermentation, the number of *S. typhimurium* continuously increased. The mechanisms of the antibacterial activity of *Lactobacillus* strains appear to be multifactorial^25^. *Lactobacillus* strains inhibit the growth of pathogenic bacteria and sometimes even kill them by lowering the pH by producing acetic and lactic acid^26^.

### Anti-adhesion effects of *L. gasseri* and *L. casei* against *S. typhimurium*

Intestinal infections initiate with the adhesion of the pathogenic bacteria to the mucosal surface. This process occurs through distinguishing the specific receptors by bacteria. Probiotic strains can adhere to gastrointestinal tract (GIT) receptors and block them from pathogens to prevent adhesion^27,28^. Competition between probiotics and pathogens for the same receptors can be a reason for balancing the GIT microbiota and preventing the infection. In this study, in the competition method after the simultaneous addition of *L. gasseri* and *S*. *typhimurium*, the adhesion rate of *S. typhimurium* decreased. The same process happened to *L. casei* to a lesser extent. Antimicrobial compounds such as hydrogen peroxide, bacteriocins, organic acids, and polysaccharides can reduce the adhesion. Other reasons include competition for nutrition and receptors^27^.

As shown in Fig. 1d, inhibitory effects on *S. typhimurium* were 44.5 and 32.9% for *L. gasseri* and *L. casei*, respectively. Competition effects on *S. typhimurium* were 54.1 and 41.6% for *L. gasseri* and *L. casei*, respectively. Currently, many studies show that *L. casei* may improve inflammatory bowel disease (IBD) by regulating the balance of intestinal microbiota and the host’s immune response and several researchers also reported that *L. casei* remarkably impedes the adhesion and invasion of pathogenic bacteria in the gut of food animals^29^. In addition to *L. casei’s* anti-adhesion and anti-inflammatory properties, it is compatible with food environment and has shown remarkable anti-diabetic properties, for example, *L. casei* NRRL-B-1922 has the high ability to grow and adapt in a *Punica granatum* juice environment, and showed acceptable antidiabetic properties^30^. Adhesion can be associated with different forms, which include the adhesion of these bacteria together or the adhesion of bacteria to host cells. An effective method is to prevent the formation of biofilm and colonization and thus prevent the initiation of infection by bacteria. Probiotic bacteria can compete nutritionally with other pathogens through the good adhesion ability to host's intestinal cells, and by bindings to the receptor, they take the chance of adhesion from the pathogens. Moreover, antimicrobial compounds such as hydrogen peroxide, which is an oxygen catabolite, and bacteriocins, which are composed of antimicrobial protein substances, as well as organic acids such as lactic acid and acetic acid, are all involved in the anti-adhesion potential^31^.

In one of the studies that related to the anti-adhesion properties of *Lacticaseibacillus rhamnosus* GG, KU200656 and *Lactiplantibacillus plantarum* KU200656, *Staphylococcus aureus* (*S. aureus*) ATCC 6538 were able to work on pathogens such as *Staphylococcus aureus*, *Listeria monocytogenes, Escherichia coli* (*E. coli*) and *Salmonella typhimurium* to have 25–78% anti-adhesion properties^31^.

Another study showed that the consumption of *L. casei* WLCA02 significantly reduced the amounts of *Salmonella* in the ileum, colon and cecum and also in the liver and spleen that are outside the intestine, and this reduction was also observed in the feces^32^.

### ABTS assay

**Figure 2:**
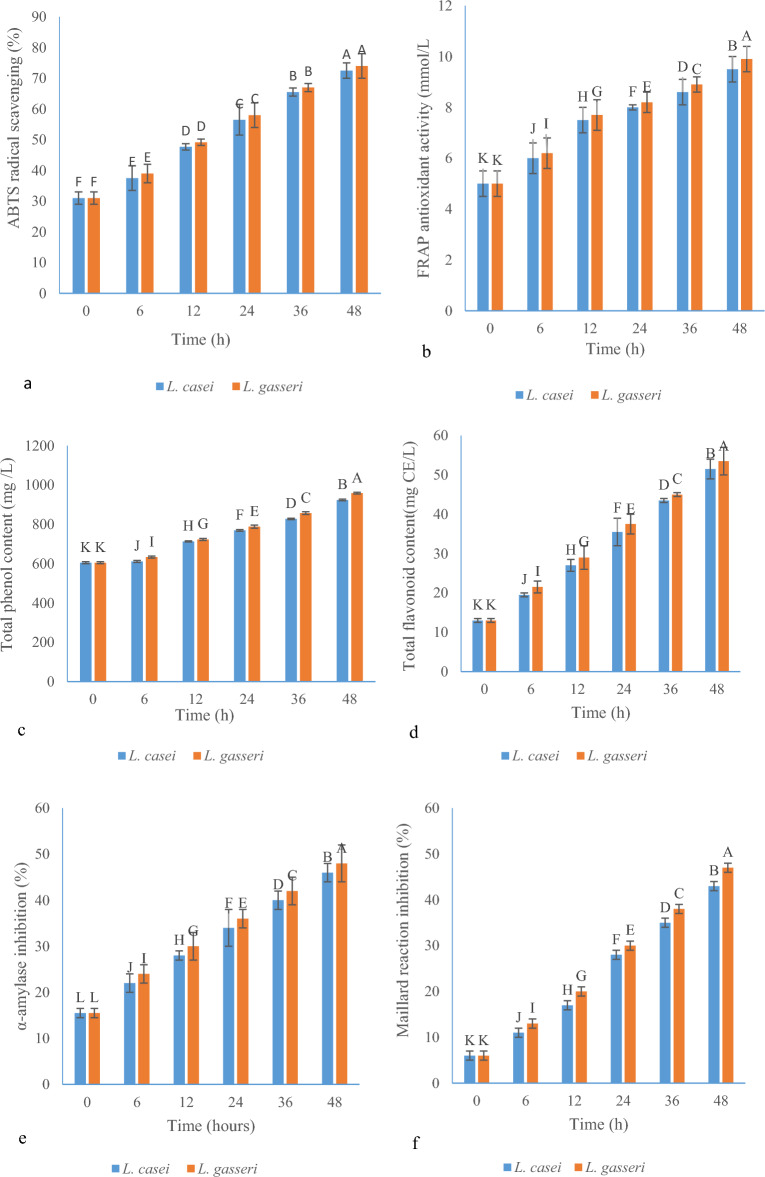
(**a**) ABTS scavenging for *L. gasseri* and *L. casei*; (**b**) antioxidant properties (FRAP) of BRJ fermented by *Lactobacillus* strains; (**c**) total phenolic content (mg/L) for *L. gasseri* and *L. casei*; (**d**) total flavonoid content (mg/L) of BRJ for *L. gasseri* and *L. casei*; (**e**) α-amylase inhibition of BRJ by *L. gasseri* and *L. casei*; (**f**) effect of *Lactobacillus* strains on Maillard reaction inhibition (%) during the fermentation of BRJ (different capital letter show the significant difference (p ≤ 0.05)).

ABTS (2,2′-azino-bis(3-ethylbenzothiazoline-6-sulfonic acid)) is a free radical used to measure antioxidant power. Phenolic compounds in fruit or vegetable juices give hydrogen to a strong oxidizing agent such as potassium persulfate in this reaction^33^. As indicated in Fig. 2a, at the beginning of fermentation, the rate of absorption of free radicals was 31% for both LABs. With the progress of fermentation, the absorption of ABTS radical for *L. gasseri* and *L. casei* strains reached 58 and 56.5% on the first day and 74 and 72.5% on the second day of fermentation of BRJ, respectively. There was a significant difference between different fermentation hours (0, 6, 12, 24, 36, and 48) in terms of antioxidant power to remove free radicals at the 5% level, but in each time of fermentation, there was no significant difference between *L. gasseri* and *L. casei* at the α = 5% level.

In 2019, the improvement of the antioxidant power of apple juice by *L. plantarum* (ATCC 14917) was investigated and the increase of antioxidant power was reported by DPPH and ABTS methods at fermentation times of 24, 48 and 72 h. The reason for this increase in antioxidant power was found to be due to the increase in the amount of phenolic and flavonoid compounds in apple juice during fermentation^34^.

### FRAP assay

The FRAP method is a process for measuring the antioxidant power based on the oxidation and reduction system. In this method, iron in the complex (Fe^III^-TPTZ) receives electrons in an acidic environment and as a result of this reaction, the color of the solution turns from yellow to blue^35,36^. The concentration of Fe^2+^ ions formed in the solution is found by measuring the absorbance at the wavelength of 593 nm. Higher absorption values in spectrophotometer indicate higher antioxidant power^37^. According to Fig. 2b, the result of the FRAP assay of BRJ at the end of 48 h of fermentation increased significantly compared to the beginning of fermentation. In this experiment, *L. gasseri* had higher antioxidant power than *L. casei* and at the end of 48 h FRAP antioxidant activities reached 9.9 and 9.5 mol/L for *L. gasseri* and *L. casei*, respectively. Similarly, in a recent study by Chen et al., *L. acidophilus* and *L. plantarum* improved the color and antioxidant activity of Strawberry during fermentation using the FRAP method^38^. At the end of 48 h of fermentation, it reached 14.54 and 14.52 mol/L for *L. acidophilus* and *L. plantarum*, respectively.

The antioxidant properties of LAB can be related to various factors, such as antioxidant enzymes resulting from their activity, exopolysaccharides and bioactive peptides produced by them, and finally manganese ions. In addition, beneficial bacteria in the intestine can produce bioactive food antioxidants using various enzymatic reactions^39^. In other researches, it is stated that LAB adapted to the environment of beverages such as jujube juice and Djulis can increase the absorption of free radicals, and thus the antioxidant properties improve through the increase of phenolic compounds^40,41^. In some studies, the effect of organic acids on antioxidant properties has been observed, such as lactic, caproic, acetic, lauric, and capric acid, which had increased significantly after the fermentation of jujube-wolfberry composite Juices and have increased the antioxidant properties^42^. Furthermore, phlorizin and caffeic acid increased antioxidant properties in apples after fermentation by LAB^43^. According to these studies, remarkable antioxidant properties were observed for LAB after the fermentation.

### Total phenolic and total flavonoid content

In BRJ, the total phenolic and flavonoid content was 605 mg/L and 13 mg CE/L, respectively (Fig. 2c,d) and both LABs were able to significantly increase the total phenolic and total flavonoid content. For example, the amount of phenolic and flavonoid compounds reached from 788 to 958.33 mg/L and 37.5 to 53.5 mg CE/L after 24 and 48 h of fermentation for *L. gasseri*, respectively. As specified in the Fig. 2c,d*, L. gasseri* was stronger than *L. casei*. For *L. casei*, the amount of phenolic and flavonoid compounds reached 923.66 mg/L and 51.5 mg CE (Catechin)/L at the end of 48 h of fermentation. These results match the previous research. Previous studies have found that fermentation can increase compounds such as kaempferol and quercetin and other phenolic and flavonoid compounds^44^. Also, the previous studies indicated the effect of fermentation time on increased phenolic compounds in okra seed, which reached the highest level (1460 mg GAE/100 g) after 24 h of fermentation (1460 mg GAE/g), while at the beginning of fermentation, it was at its lowest (185 mg GAE/100 g)^45^.

There are different opinions about the increase in the amount of phenolic compounds due to the fermentation of LAB, but what is common among the theories is the activity of enzymes secreted by LAB. For example, in one study, *Lactobacillus* could produce a wider range of phenolic acids through ring fission, deglycosylation, reductive metabolism of phenyl acyl fragments, aromatic dehydroxylation and hydroxylation, decrease of double bonds (carbon–carbon) and all these operations were performed on anthocyanins^38^. In addition, in the fermentation process by *Lactobacillus* in Jussara pulp, secreted β-galactosidase and α-galactosidase enzymes were involved in the destruction and conversion of anthocyanins^46^.

In addition, in another research, it was reported that as a result of the activity of enzymes that are released in the fermentation process, compounds such as tannins, flavonoids, phenylpropanoid, and alkaloids are produced in a greater amount. In the fermentation process, LAB polymerizes complex phenolic compounds and converts them into simple phenolic compounds. It has been mentioned in a study that natural fermentation using microorganisms increases acidity and β-glucosidase produced by *L. plantarum* via the hydrolysis of complex phenolic compounds that enable them to produce simple phenolic compounds in high amounts^47^.

### HPLC analysis

HPLC analysis of phenolic compounds of BRJ after 48 h of fermentation by *L. gasseri* is shown in Table 1. Among these 10 compounds, anthocyanins have a significant contribution in terms of quantity. Ellagic acid and cyanidin 3-glucoside had the highest amount among phenolic compounds and chlorogenic acid had the lowest amount. The order of phenolic compounds was as follows:

**Table 1:** The polyphenolic compounds in fermented black raspberry juice by *L. gasseri*.

Entry	Compound name	Retention time (min)	Height (mA U)	Sample concentration (mg/100 g)
1	Gallic acid	7.55	192.047	11.1095
2	Chlorogenic acid	11.950	32.778	0.30569
3	Myricetin 3-glucoside	13.767	30.771	0.91096
4	Cyanidin 3-glucoside	14.400	367.529	19.9755
5	Kaempfrol	14.833	74.238	4.4367
6	*p*-Coumaric acid	15.617	39.687	2.5241
7	Caffeic acid	16.533	121.334	7.75
8	Elagic acid	17.467	386.853	33.695
9	Ferulic acid	18.367	132.591	10.58
10	Quercetin	23.033	111.992	4.5241
	Total		1489.821	95.81155

Ellagic acid > Cyanidin 3-glucoside > Gallic acid > Ferulic acid > Caffeic acid > Quercetin > Kaempfrol > *p*-Coumaric acid > Myricetin 3-glucoside > Chlorogenic acid.

Finally, the results of this study are consistent with the previous studies, as it has been reported that ellagic acid had the highest amount (2875 mg/kg) among other phenolic compounds of Raspberry leaf extract^48^.

### α*-Amylase inhibition*

Compounds that inhibit the α-amylase enzyme prevent the increase of blood glucose and even reduce it after eating. In fact, they do this by slowing down the breakdown of carbohydrates in the small intestine and reducing postprandial hyperglycemia^49^. As seen in Fig. 2e, at the beginning of fermentation, the inhibition rate of the α-amylase enzyme was 15.5%, and with the start of the fermentation process by two LABs, the inhibition rate increased to a greater extent. At the end of 48 h of fermentation, the inhibition rate reached 48 and 46% for *L. gasseri* and *L. casei* bacteria, respectively. α-Amylase inhibitors prevent the absorption of starch from foods in the body, so-called a starch blocker. Because starch is a carbohydrate with a complex structure, it is not easily absorbed by the body unless it is broken down by α-amylase or other related enzymes^50^.

In previous studies, it has been stated that the inhibition of α-amylase by *Morindalucida* leaf extract may be due to the presence of several compounds present in the plant such as tannins, flavonoids and saponins^49^. In another study, α-amylase and α-glucosidase inhibitory activity of six groups of flavonoids were investigated and it was found that from the category of flavonols, myristin 64%, Quercetin 50%, Kaempferol 18% and Cyanidins from the category of anthocyanins inhibited α-amylase by more than 50%^51^. Therefore, due to the presence of the above compounds in the sample of FBRJ, the increase in inhibition of α-amylase can be related to the flavonols and anthocyanins of BRJ.

### Maillard reaction

The Maillard reaction occurs between amino acids and reducing sugars, and brown aromatics compounds and UV-absorbing intermediates are the end products of this reaction^52,53^. LAB converts sugars into organic acids such as acetic, propionic, and lactic acids^54^ which leads to an increase in the ratio of non-reducing sugars to reducing sugars in the entire fruit juice environment, thus, the needed primary substance to carry out the Maillard reaction is removed, and the reaction is prevented^55^. Previous studies have also shown that fructose and glucose levels decreased during fermentation due to the activity of LAB and the consumption of simple sugars^55,56^. In the present work, *L. gasseri* and *L. casei* were able to prevent the Maillard reaction by 47 and 43%, respectively during 48 h of fermentation (Fig. 2f), while in the first of the fermentation, this amount was 6% in BRJ. Compared to the initial time of fermentation, the prevention of Maillard reaction was more effective in the first 24 h of the fermentation than in the second 24 h of the fermentation.

### Antibacterial activity

In this study, three samples called fermented black raspberry juice (FBRJ), black raspberry juice (BRJ) and black raspberry Juice + *Stevia* extract (BRJ + SE) were used. All results were reported in terms of growth inhibition area (mm) in Table 2. All samples had antibacterial properties. *E. coli* were more sensitive than *S. aureus* to the samples and a larger inhibition zone was obtained (Table 2). The highest antibacterial power was related to BRJ + SE (25 g/L) against *E. coli* with diameters of 27 and 28 mm and the lowest was related to BRJ against *S. aureus* with a diameter of 6.7 and 7 mm, in VAT and HHP processing, respectively. The important point is that due to the fermentation process, the antibacterial activity of all fermented samples by *L. gasseri* and *L. casei* increased compared to unfermented samples. These results are consistent with the antibacterial properties of Fuji” apple juice. In that research, it was reported that samples fermented with *L. acidophilus, L. casei* and *L. plantarum* had higher antibacterial power than unfermented samples^57^.

**Table 2:** Antibacterial activity of fermented black raspberry Juice (FBRJ), black raspberry juice (BRJ) and black raspberry Juice + *Stevia* extract (BRJ + SE).

Sample juice	Mean zone of inhibition (mm)	
	Bacterial strain	
	*E. coli*	*S. aureus*
FBRJ (*L. gasseri*)	17.5 ± 0.3	11.9 ± 0.4
FBRJ (*L. casei*)	16.5 ± 0.5	9.5 ± 0.11
BRJ (VAT)	9.7 ± 0.2	6.7 ± 0.3
BRJ (HHP)	10.7 ± 0.26	7 ± 0.4
BRJ + SE (25 g/L) (VAT)	27 ± 0.35	14.5 ± 0.36
BRJ + SE (25 g/L) (HHP)	28 ± 0.26	15.5 ± 0.5

### Pasteurization processes and *Stevia* extract on the stability of phenolic and flavonoid compounds

**Figure 3:**
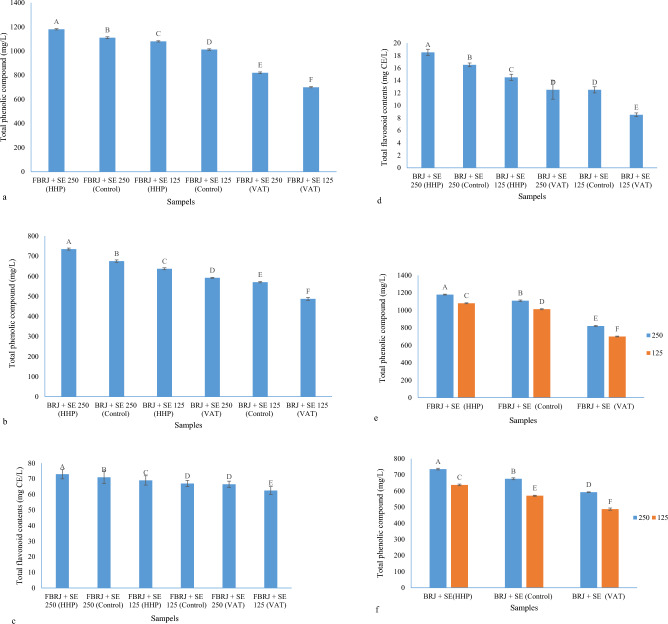
(**a**) Total phenolic compounds of FBRJ samples combined with *Stevia* extract on the 28th day; (**b**) total phenolic compounds of unfermented BRJ samples combined with *Stevia* extract on the 28th day; (**c**) total flavonoid contents of FBRJ samples combined with *Stevia* extract on the 28th day; (**d**) total flavonoid contents of unfermented BRJ samples combined with *Stevia* extract on the 28th day; (**e**) simultaneous effect of concentration and pasteurization process on phenolic compounds (FBRJ) after 28 days; (**f**) simultaneous effect of concentration and pasteurization process on phenolic compounds (unfermented BRJ) after 28 days (different capital letters show the significant difference (p ≤ 0.05)).

*Stevia* extract was added at the rate of 12.5 and 25 g/L to the BRJ samples and pasteurization processes such as HHP and VAT were applied to the samples. The amount of phenolic and flavonoid compounds was measured on the 28th day and the amount of reduction of these compounds after 28 days was also calculated to determine the exact effect of extract and pasteurization processes on phenolic and flavonoid compounds. As shown in Fig. 3a,b, all fermented and unfermented raspberry samples mixed with *Stevia* extract at two levels of 12.5 and 25 g/L and subjected to different pasteurization processes have a significant difference from each other on day 28 in terms of the amount of phenolic compounds. The fermented and unfermented sample of BRJ that was mixed with *Stevia* extract at a level of 25 g/L and subjected to the HHP process (FBRJ + SE 25 (HHP)) and (BRJ + SE 25 (HHP)) was the highest in terms of the amount of phenolic compounds, and the fermented and unfermented sample of BRJ, which was mixed with *Stevia* extract at the level of 12.5 g/L and was subjected to VAT process (FBRJ + SE 12.5 (VAT)) and (BRJ + SE 12.5 (VAT)) were the lowest in terms of phenolic compounds. The order of the values of phenolic compounds is shown in Fig. 3a,b.

Regarding the remaining flavonoid compounds after 28 days, the results were according to phenolic compounds with slight differences. There was a significant difference between all samples, except FBRJ + SE 12.5 (Control) and FBRJ + SE 25(VAT) samples, as well as between BRJ + SE 25 (VAT) and BRJ + SE 12.5 (Control) samples that there was no significant difference between the mentioned samples. There was also no significant difference in terms of quantity and the last-mentioned samples were even completely equal in terms of quantity (Fig. 3c,d).

The order of strength of samples for flavonoid contents is as follows:

$${\text{FBRJ}} + {\text{SE}}\;25\;({\text{HHP}}) > {\text{FBRJ}} + {\text{SE}}\;25\;({\text{Control}}) > {\text{FBRJ}} + {\text{SE}}\;12.5\;({\text{HHP}}) > {\text{FBRJ}} + {\text{SE}}\;12.5\;({\text{Control}}) > {\text{FBRJ}} + {\text{SE}}\;25\;({\text{VAT}}) > {\text{FBRJ}} + {\text{SE}}\;12.5\;({\text{VAT)}}$$

The simultaneous effect of concentration and pasteurization process on phenolic compounds after 28 days can be seen in Fig. 3e,f, and the HHP process is the best process for pasteurization of fermented and unfermented samples of BRJ with *Stevia* extracts at different levels. The VAT pasteurization sample was so destructive that even the amount of phenolic compounds remained in this method was lower than the control sample, and these amount at the end of 28 days for the fermented samples that were combined with 25 and 12.5 g/L of *Stevia* were 820 and 700 mg/L, respectively and in the HHP process 1180, 1080 and 1110 and 1012 mg/L were reported for the control sample, which indicates the positive effect of the HHP process and the negative effect of the VAT process on the pasteurization of phenolic compounds. The same trend prevailed for non-fermented samples; the only difference was that the non-fermented samples that were combined with *Stevia* extract were at a lower level than the non-fermented samples in terms of the amount of remaining phenolic compounds. For BRJ + SE 25 (HHP) and BRJ + SE 12.5 (HHP) and BRJ + SE 25 (Control) and BRJ + SE 12.5 (Control) and BRJ + SE 25 (VAT) and BRJ + SE 12.5 (VAT), respectively, the values of 735, 675, 637, 592, 570, 487 mg/L were obtained and as it is known, the higher concentration of *Stevia* extract is more reliable in the stability of phenolic compounds than lower concentrations. The same effect was seen for flavonoid compounds. These results are consistent with the results of Rios-Corripio et al. They investigated the HHP process with different pressures and times for the stability of phenolic and flavonoid compounds and anthocyanins of fermented pomegranate juice. They compared the HHP process with VAT (63 °C/30 min) and HTST (72 °C/15 s) methods, as well as the control. HHP method, HTST, control and VAT were the best to worst pasteurization processes to preserve phenolic and flavonoid compounds and anthocyanins of fermented pomegranate juice, respectively^58^.

**Figure 4:**
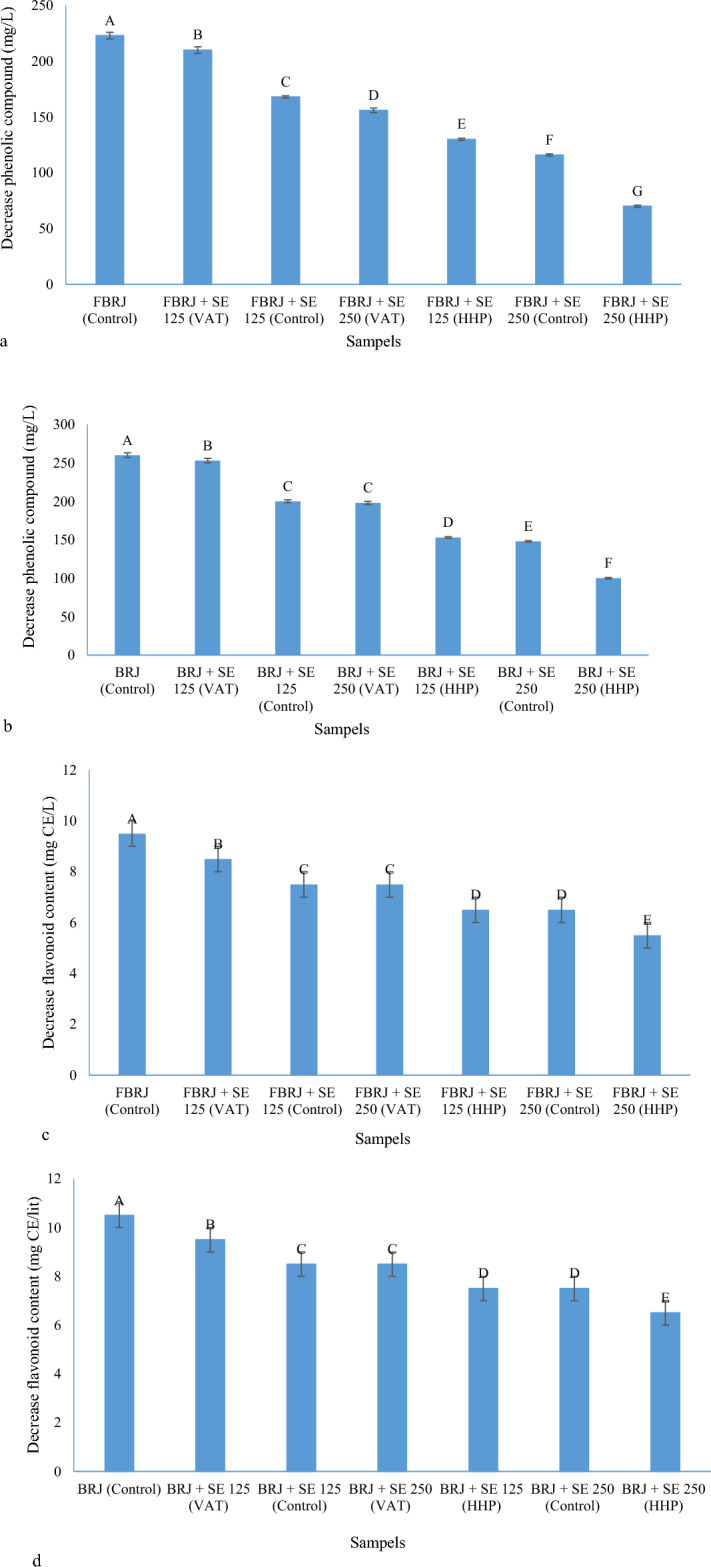
(**a**) Decrease phenolic compound for FBRJ samples during 28 days; (**b**) decrease phenolic compound for unfermented BRJ samples during 28 days; (**c**) decrease flavonoid content for FBRJ samples during 28 days; (**d**) decrease flavonoid content for BRJ samples during 28 days (different capital letters show the significant difference (p ≤ 0.05)).

As shown in Fig. 4a–d, the amount of reduction of phenolic and flavonoid compounds for fermented and non-fermented samples of BRJ without adding *Stevia* extract is higher than in other samples. For example, for the sample fermented by *L. gasseri* and the non-fermented sample to which no extract was added, the reduction of phenolic compounds was observed by 223 and 260 mg/L, respectively, which was the largest reduction among the samples. For the fermented and non-fermented samples of BRJ to which 25 g/L of *Stevia* extract was added and subjected to the HHP process, 70 and 100 mg/L reduction of phenolic compounds was observed, respectively, which was the lowest decrease among the samples. Also, in the case of flavonoids, a decrease in flavonoid compounds was observed by 9.5 and 10.5 mg CE/L, respectively, for the sample fermented by *L. gasseri* and non-fermented to which no extract was added, which had the largest decrease among the samples. For the fermented and non-fermented samples of BRJ to which 25 g/L of *Stevia* extract was added and subjected to HHP process, 5.5 and 6.5 mg/L reduction of flavonoid compounds was observed, which was the lowest reduction among the samples. It can be concluded that increasing the concentration of *Stevia* extract along with the HHP process can help the stability of phenolic and flavonoid compounds after 28 days of storage in the refrigerator. In a study, the stability of phenolic, antioxidant, and antidiabetic compounds of roselle drink was investigated using *Stevia* and citric acid; It was reported that the addition of *Stevia* to the beverage increased the stability of gallic acid, rosmarinic acid, epigallocatechin gallate, and quercetin during storage for 12 days. It was also reported about anthocyanins that the content of total anthocyanin after storage had a slight increase in the content of pelargonidin-3-glucoside, cyanidin-3-glucoside, and peonidin-3-glucoside after storage and these compounds are pigments that have an important effect in increasing the antioxidant property in fruit juice and over time due to the changes that occur in the B-ring of anthocyanins compounds, 3,4-dihydroxybenzoic and phydroxybenzoic, 3,4,5-trihydroxybenzoic acids are produced and one of the characteristics of the produced compounds is that they are hydrogen donors and inhibit the activity of free radicals and thus increase the antioxidant activity. In addition, they increase the amount of total phenolic compounds during storage^59^. According to the above, for this research, such a result can be taken in relation to the changes in anthocyanins and the increase of phenolic acids in BRJ.

In addition, in previous research conducted on *Stevia*, it has been determined that this plant has many glycosides and is mainly composed of steviol, which can link with the surrounding sugars or glycosides with these glycosidic bonds^60^. Therefore, it can have a protective role on the phenolic compounds of BRJ over time. Another study showed that glycosides and pigments in *Stevia* have been preserved to a significant extent even after bread processing and have preserved the antioxidant property of bread^61^; therefore, *Stevia* has many pigments that can bond with the pigments of BRJ and create a protective complex.

It has been reported that phenolic compounds extracted from plants have medicinal properties, including anti-diabetic properties and anti-inflammatory properties^62,63^. It has also been reported that the extraction of phenolic compounds in three forms, free, esterified and insoluble-bound, from (*Cucumaria frondosa*) tentacles significantly control the amount of secondary oxidation products in fish^64^. On the other hand, raspberries have valuable terpenes such as α-pinene, β-myrcene and linalool, and among these compounds^65^, it has been reported that α-pinene, in addition to having therapeutic properties such as protecting the heart and being anti-cancer, also has flavoring properties, so the extract and essential oil of this fruit together with *Stevia* can be used as a natural preservative in food for a long time^66^.

## Conclusion

In conclusion, fermentation by LAB is an effective method to increase the biological properties of BRJ with low concentration and both *L. gasseri* and *L. casei* bacteria can have a significant effect on the growth inhibition of *S*. *typhimurium* and its adhesion to intestinal cells. In addition, the results show that the amount of total phenolic compounds such as gallic acid, cinnamic acid, and ferulic acid increases due to the addition of LAB to BRJ. Subsequently, it was found in the antioxidant tests that the antioxidant power of BRJ has also increased significantly. Also, the increase in the FRAP power and ABTS scavenging assay and α-amylase inhibition caused by these two LABs in BRJ made it a special product, and *L. gasseri* was more effective than *L. casei* in increasing the biological properties of fruit juice. Considering the reduction of natural sugars in BRJ, due to fermentation and the sweetening properties, and high antioxidant and antibacterial power of *Stevia* extracts, adding *Stevia* extract to FBRJ and performing the HHP process to protect as much phenolic compounds as possible on the resulting mixture can create an excellent product in terms of phenolic and flavonoid compounds which can maintain these biological properties to an acceptable level for up to 28 days. Also, considering the survival of these two bacteria after 48 h of fermentation in fruit juice and also considering the dairy origin of these two bacteria, they can be a suitable option for use in food products, especially dairy products. Protection of increased phenolic and flavonoid compounds due to the fermentation of *L. gasseri* and *L. casei* bacteria in BRJ during 28 days of storage at refrigerator temperature is a new method that has not been carried out so far. The combination of *Stevia* plant extract and the use of the appropriate HHP method in fermented and non-fermented BRJ, and obtaining positive results regarding the protection of the phenolic compounds of BRJ in a long period of preservation are the significant findings of this study that have not been reported so far.

## Experimental

### Materials and instruments

*Lactobacillus gasseri SM 05*, *Lacticaseibacillus casei* subsp*. casei* (PTCC 1608) and *Salmonella enterica* subsp*. enterica* serotype *typhimurium* (PTCC 1709) were purchased from the Institute of Industrial Fungi and Bacteria of Iran. *Caco-2 cells* and *Lactobacillus* strain were prepared according to Behbahani et al*.*^27^. The pH was measured with a pH meter (WTW Bench pH meter 7110, Germany). High performance ternary gradient liquid chromatography system was used for the identification of major phenolic compounds (Azura model from Knauer Co. made in Germany), equipped with P6.1L pump and MWD 2.1 UV detector.

### Preparation and fermentation of black raspberry juice

Plant sample collection was in accordance with relevant institutional, national, and international guidelines and legislation. Collection of raspberry samples was according to IUCN policy statement on research involving species at risk of extinction. As it was not a conserved species, permission for collection and also growth location of the plant was obtained from the agricultural office of Guilan province and the minimum necessary amount was harvested. After the collection of raspberry samples, waste materials were separated from each fruit. It is important to note that the used species from BRJ in this work was *R. dolichocarpus*. The fruit was washed, homogenized, and filtered through sterilized gauze to obtain the juices. BRJ was prepared according to Li et al.^24^ with slight modifications. The pH of the juice was adjusted to about 4 with 1 M Na_2_CO_3_. The amount of Brix in the juice was adjusted to 15, and then the juice was pasteurized at 80 °C for 5 min. In the next stage, the inoculation of juice was started with different bacteria (1% v/v). The initial microbial count was approximately 5.5 log colony forming units (CFU)/mL. Juice samples inoculated with bacteria were kept in the dark at 37 °C for 48 h and following biological tests were performed at the intervals of 0, 6, 12, 24, 36, and 48 h. To evaluate the activity of LAB and pathogens in the fruit juice, the following was done: *L. gasseri*, *L. casei* and *S. typhimurium* were added to juice at about 5.5 log CFU/mL. The control sample was BRJ containing *Salmonella* without LAB that incubated at 37 °C for 48 h and then the bacteria were counted.

### Extraction of *Stevia* extract

*Stevia rebaudiana* Bertoni was obtained from the farms of Guilan province and *Stevia* extract was obtained using the extraction method^67^ by pure water solvent. After extraction, the extract was concentrated and used fresh to protect the phenolic compounds of BRJ within 28 days. Some experiments including measurement of total phenolic and total flavonoid compounds, and antibacterial activity were performed on *Stevia* extract in combination with BRJ.

### Determination of viable cells

Viable cell counts were obtained by serial dilution with sterile peptone water until 10^–6^ dilution. Aliquots of 0.1 mL of dilution were plated, in triplicate in plates containing MRS Agar (spread plate method). The plates were incubated for 48 h at 30 °C. Plates containing 30–300 colonies were measured and recorded as colony forming units (CFU) per 1 mL of solution^68^.

### Anti-adhesion activity of *L. gasseri* and *L. casei* against *S. typhimurium*

This experiment was done with two approaches: competition and inhibition. In the competition approach, strains of *L. gasseri* and *L. casei* and *S. typhimurium* were added to Caco-2 cells simultaneously (in equal proportions) and incubated for 1 h and the unbound bacteria were washed with sterilized PBS. Then, this work was done once without LAB and the percentage of competition for adhesion was calculated according to the formula of Behbahani et al.^27^.

In the inhibition approach, the first two LABs were incubated for 1 h after being added to Caco-2 cells and the unbound bacteria were washed with sterilized PBS immediately *Salmonella* was added to the container and after 1 h of incubation, the washing step was carried out. After that, the cells and bacteria were separated with Triton (X-100, 0.05%), and the percentage of inhibition of its adhesion was calculated according to the formula of Behbahani et al.^27^.

### ABTS radical scavenging assay

ABTS assay of BRJ was prepared according to the literature^34^. Briefly, 2.45 mM potassium persulfate was mixed with 7 mM ABTS solution, and the mixture was kept at 25 °C for 12 h before use. The dilution of ABTS^**·**+^ solution was prepared with ethanol and obtained an absorbance of 0.70 at 734 nm. Next, 0.3 mL from samples of BRJ reacted with diluted ABTS^**·**+^ solution (5 mL) and absorbance were read after six min at 734 nm. Then, RSA was calculated according to the following formula:

$$\mathrm{ABTS\, RSA }(\mathrm{\%}) =[({\text{A}}0-{\text{As}})/{\text{A}}0] \times 100$$

where A0 is the absorbance of ABTS radical solution without sample and As is the absorbance of the sample^34^.

### FRAP assay

Briefly, at first FRAP reagent (1.8 mL) was blended with distilled water (0.2 mL), then the solution was mixed with 50 µL BRJ and incubated for 10 min at 37 °C and immediately the absorbance of the resulting mixture was read at 593 nm by spectrophotometer based on mol of Fe^2+^/L^69^.

### Total phenolic content

Specifically, 0.2 mL samples (BRJ during fermentation) or (BRJ + *Stevia*) with proper dilution were mixed with 1.5 mL of tenfold-diluted Folin-Ciocalteu reagent and 1.5 mL of 7.5% (w/v) sodium carbonate. After holding at room temperature in darkness for 40 min, the absorbance was read at 765 nm using a UV–Vis spectrophotometer. The total phenolic content was standardized against Gallic acid and expressed as mg Gallic acid equivalents/L^24^.

### Total flavonoid content

Determination of total flavonoid content was performed according to Salarbashi et al. method. Total flavonoid content was expressed as mg catechin equivalents/L^70^.

### High performance liquid chromatography (HPLC) analysis

For this purpose, 2 mL of BRJ was transferred to a 10 mL flask and made up to 10 mL with methanol. The mixture was homogenized for 20 min at room temperature in an ultrasonic bath. Then, it was centrifuged for 15 min at a speed of 5000 rpm. The clear methanolic solution was passed through a 0.45-µm filter and then used for analysis with the HPLC device according to the below conditions. The chromatographic system comprised a C18 column (Zorbax Extend-C18, 5 μm, 250 × 4.6 mm, Agilent). Mobile phase: acetonitrile, Buffer: 0.5% v/v acetic acid, injection volume: 20 µL and pump program: start from 10% acetonitrile, rising to 100% during 35 min. The flow rate was 1 mL/min and diode-array detection was performed at 280 nm^71^.

### α-Amylase inhibition

The α-amylase solution was obtained according to the literature^72^. Briefly, 1 mL of BRJ samples were mixed with starch solution (3.5 mL, 1%), α-amylase solution (4 mL), 3,5-dinitrosalicylic acid and distilled water (10 mL) and incubated for 15 min at 30 °C. The reaction stopped by sodium carbonate (200 µL, 0.1 M). The absorbance was read at 540 nm. The α-amylase inhibitory activity was calculated using the formula:

$$\mathrm{\%inhibition }= (\mathrm{A \,control }-\mathrm{ A \,sample}/\mathrm{A \,control}) \times 100$$

### Maillard inhibition

Inhibition of Maillard reaction was measured based on Hashemi et al*.*^55^.

### Antibacterial activity

First, for fermented samples, the filtration of fermented juice was performed by a nominal 0.22 mm filter. Then, 20 µL of the BRJ or *Stevia* extract (25 g/L) combined with BRJ was poured on sterile disks (6 mm diameter) and placed on the Muller-Hinton agar containing 10^7^ bacterial cells per mL under aseptic conditions. Afterward, the plates were incubated (37 °C and 24 h). After incubation, the diameter around the disks (zones of inhibition) was measured^73,74^.

### Effect of pasteurization processes and *Stevia* extract on the stability of phenolic and flavonoid compounds

Different processes were used to measure the stability of phenolic compounds during 28 days. At first, the HHP process was used according to Cantúa et al. method, and based on the results obtained in relation to phenolic compounds in this study, a pressure of 600 MPa was used for 10 min at room temperature^75^. Also, the pasteurization method called VAT (63 °C/30 min) was used^58^. In addition to using these processes, the extract of *Stevia rebaudiana Bertoni* plant at two levels of 12.5 and 25 g/L was also used to evaluate the stability of phenolic compounds in different samples of BRJ (fermented and unfermented).

### Statistical analysis

The data were presented as the mean ± standard deviation (SD). All the experiments were performed in triplicate. The one-way analysis of variance (ANOVA) was used to compare the means by SAS software (version 9.4). To evaluate whether the fermented BRJ samples were different from the control, in most assays, their difference percentage was calculated and significant differences between the two fermented juices were obtained using the student t-test. A significant level was considered as p value ≤ 0.05.

## Data Availability

The datasets used and/or analyzed during the current study are available from the corresponding author on reasonable request.
